# Trends in Opioid Use in Pediatric Patients in US Emergency Departments From 2006 to 2015

**DOI:** 10.1001/jamanetworkopen.2018.6161

**Published:** 2018-12-21

**Authors:** Daniel M. Tomaszewski, Cody Arbuckle, Sun Yang, Erik Linstead

**Affiliations:** 1 School of Pharmacy, Department of Biomedical and Pharmaceutical Sciences, Chapman University, Irvine, California; 2 Schmid College of Science and Technology, Mathematics and Computer Science, Chapman University, Orange, California; 3 Department of Pharmacy Practice, Chapman University School of Pharmacy, Irvine, California

## Abstract

**Importance:**

The use of opioids to treat pain in pediatric patients has been viewed as necessary; however, this practice has raised concerns regarding opioid abuse and the effects of opioid use. To effectively adjust policy regarding opioids in the pediatric population, prescribing patterns must be better understood.

**Objective:**

To evaluate opioid prescribing patterns in US pediatric patients and factors associated with opioid prescribing.

**Design, Setting, and Participants:**

This cross-sectional study used publicly available data from the National Hospital Ambulatory Medical Care Survey from January 1, 2006, to December 31, 2015. Analysis included the use of bivariate and multivariate models to evaluate factors associated with opioid prescribing. Practitioners from emergency departments throughout the United States were surveyed, and data were collected using a representative sample of visits to hospital emergency departments. The study analyzed all emergency department visits included in the National Hospital Ambulatory Medical Care Survey for patients younger than 18 years. All statistical analysis was completed in June of 2018 and updated upon receiving reviewer feedback in October of 2018.

**Exposures:**

Information regarding participants’ medications was collected at time of visit. Participants who reported taking 1 or more opioids were identified.

**Main Outcomes and Measures:**

Evaluation of opioid prescribing patterns across demographic factors and pain diagnoses.

**Results:**

A total of 69 152 visits with patients younger than 18 years (32 727 female) were included, which were extrapolated by the National Hospital Ambulatory Medical Care Survey to represent 293 528 632 visits nationwide, with opioid use representing 21 276 831 (7.25%) of the extrapolated visits. Factors including geographic region, race, age, and payment method were associated with statistically significant differences in opioid prescribing. The Northeast reported an opioid prescribing rate of 4.69% (95% CI, 3.69%-5.70%) vs 8.84% (95% CI, 6.82%-10.86%) in the West (*P* = .004). White individuals were prescribed an opioid at 8.11% (95% CI, 7.23%-8.99%) of visits vs 5.31% (95% CI, 4.31%-6.32%) for nonwhite individuals (*P* < .001). Those aged 13 to 17 years were significantly more likely to receive opioid prescriptions (16.20%; 95% CI, 14.29%-18.12%) than those aged 3 to 12 years (6.59%; 95% CI, 5.75%-7.43%) or 0 to 2 years (1.70%; 95% CI, 1.42%-1.98%). Patients using Medicaid for payment were less likely to receive an opioid than those using private insurance (5.47%; 95% CI, 4.79%-6.15% vs 9.73%; 95% CI, 8.56%-10.90%). There was no significant difference in opioid prescription across sexes. Opioid prescribing rates decreased when comparing 2006 to 2010 with 2011 to 2015 (8.23% [95% CI, 6.75%-9.70%] vs 6.30% [95% CI, 5.44%-7.17%]; *P* < .001); however, opioid prescribing rates remained unchanged in specific pain diagnoses, including pelvic and back pain.

**Conclusions and Relevance:**

This research demonstrated an overall reduction in opioid use among pediatric patients from 2011 to 2015 compared with the previous 5 years; however, there appear to be variations in factors associated with opioid prescribing. The association of location, race, payment method, and pain diagnoses with rates of prescribing of opioids suggests areas of potential quality improvement and further research.

## Key Points

**Question:**

How has opioid use in pediatric patients changed over the past 10 years, and what factors are associated with differences in opioid use?

**Findings:**

A cross-sectional study of 69 152 emergency department visits found that opioid prescribing rates decreased between 2006 to 2010 (8.23%) and 2011 to 2015 (6.30%). Region, race, age, and payment method were associated with differences in opioid prescribing.

**Meaning:**

Opioid rates have decreased in recent years but inconsistencies in prescribing continue, suggesting the need for further research.

## Teaser

This cross-sectional study examines patterns of opioid prescription to US pediatric patients during visits to hospital emergency departments between 2006 and 2015 using data from the National Hospital Ambulatory Medical Care Survey.

## Introduction

Acute pain is common among pediatric patients presenting to hospital emergency departments (EDs).^1^ The cause of pediatric pain disorders includes, but is not limited to, headaches, abdominal pain, back pain, musculoskeletal injury, postoperative pain, and cancer-related pain. In the treatment of moderate and severe acute pediatric pain, it can be difficult to balance the risks of treatment while providing adequate pain control. Health care practitioners often must choose between the use of opioids, traditional nonnarcotic agents (including nonsteroidal anti-inflammatory drugs and acetaminophen), and nonpharmacological options.

The use of opioids to treat moderate to severe pain in pediatric patients for specific pain disorders has generally been considered appropriate and necessary; however, recent concerns regarding opioid abuse and increases in opioid-related hospitalizations and deaths among pediatric patients have raised questions about the practice of prescribing opioids for management of pain in children.^2^ Although not as extensive as in adults, the use of opioids in pediatric patients had seen a significant increase prior to 2010, with the rate of opioid prescribing doubling from 1994 to 2007 in pediatric patients.^3^ More specifically, ED prescribing of opioids in pediatric patients increased from occurring in 11.2% of visits in 2001 to 14.5% in 2010.^4^ Additionally, studies^5^ suggest that when opioids are prescribed to pediatric patients, approximately 60% of opioid prescriptions dispensed include more opioids than are needed to treat the acute pain disorder.

Recent studies^6^ have shown that as opioid prescribing has increased in pediatric patients, the rate of opioid abuse and opioid-related death have also increased among these patients. Additionally, in recent years, as opioid prescribing rates have actually declined among pediatric patients, opioid-related hospitalizations have continued to increase.^7^ This continued trend reinforces the potential risk associated with opioid use in pediatrics, with increased likelihood and accelerated progression from use to abuse in pediatric populations.^8^ Research has shown that young people who report receiving a legitimate prescription for a narcotic pain medication by 12th grade are significantly more likely to abuse prescription pain medications in the future.^9,10^ This risk for misuse has also been shown among pediatric patients who report nonmedical use of opioids prior to adulthood.^11^ The risks associated with use of opioids at an early age suggest the need for further review of opioid prescribing in pediatric patients.

This study aims to evaluate trends of pediatric opioid prescribing in EDs in US hospitals and patient-related factors associated with opioid prescribing.

## Methods

This study analyzes data collected from the 2006 to 2015 National Hospital Ambulatory Medical Care Survey (NHAMCS) focusing on patients younger than 18 years. This survey is conducted using a representative sample of visits to EDs, collecting data from medical records on patients’ symptoms, diagnoses, comorbidities, demographic characteristics, and medications ordered or provided. The survey uses a complex 4-stage study design. A description of the sampling design is available elsewhere.^12,13^ Prior to completion of this study, the Chapman University institutional review board reviewed and granted this study exempt status, as the analysis of deidentified, publicly available data does not constitute human subjects research as defined in federal regulations and as such does not require review. Hence, informed consent was not necessary. The study followed the Strengthening the Reporting of Observational Studies in Epidemiology (STROBE) reporting guideline.

The NHAMCS data were weighted by the Centers for Disease Control and Prevention (CDC) using the most recent census data to most accurately represent national hospital visit and patient population characteristics. All analyses were conducted on weighted data, as recommended by the CDC’s National Center for Health Statistics, to be representative of national hospital visit characteristics. The use of opioids was determined using the CDC’s New Ambulatory Care Drug Database system, with drugs being classified as *opioid* or *other*.^14^ Medications included all drugs reported as either ordered as a prescription or provided during the visit.

A visit was considered pain related if a pain diagnosis was reported in the reasons for the visit through the evaluation of reported *International Classification of Diseases, Ninth Revision, Clinical Modification* codes. Pain diagnosis codes were established based on the clinical classification software categories available from the Agency for Healthcare Research and Quality Healthcare Cost and Utilization project. Included pain categories were abdominal pain, arthritis or joint pain, back pain, cancer-related pain, chest pain, cholelithiasis, fractures, headache, injury excluding fracture, neck pain, nephrolithiasis, pelvic pain, sickle cell anemia, dental or jaw pain, fibromyalgia, and peripheral neuropathy. Pain categories that did not meet the minimum of 30 visits in the unweighted sample were recategorized into a category labeled *other pain*.

Metropolitan setting of the visit was determined using Metropolitan and Micropolitan Statistical Area methodology, the definition used by federal agencies for research purposes. Length of visit was recoded into dichotomous categories of prolonged (lasting >240 minutes) or not prolonged (lasting ≤240 minutes).

### Statistical Analysis

All participants’ records were stored in a relational database using the open-source database software MySQL version 5.7.11 (Oracle). All statistical analyses were preformed using the open-source statistical computing software R version 3.2.3 (R Foundation) and finalized October 12, 2018. The function *svydesign* from the R package survey was used to account for stratified, clustered, and weighted variables in the NHAMCS data. A Wald test of association was used to determine significance for all analyses.

Descriptive statistics were used to compare the characteristics of ED visits associated with opioids with those in which patients were not currently prescribed an opioid. Wald tests of association were used to determine significance for bivariate analysis, and logistic regression models were conducted using weighted patient data with opioid use as the dependent variable and age, race, sex, pain diagnosis, payment method, region, and length of ED visit as dependent variables. The model was simultaneously adjusted for all dependent variables.

All confidence intervals correspond to 95% CIs, as computed from the standard error. Comparison and associations were considered significant if 2-sided *P* < .05. All analysis conducted used cohorts of more than 30 visits and had a relative standard error of less than 30% per CDC guidelines. The CDC provided detailed documentation of the NHAMCS instrument, methodology, and data files that were used as the basis for this analysis and are available elsewhere.^12^

## Results

A total of 69 152 hospital ED visits by patients younger than 18 years at the time of the visit (32 727 female) from January 1, 2006, through December 31, 2015, were included. These visits were extrapolated by the National Hospital Ambulatory Medical Care Survey to represent 293 528 632 hospital ED visits throughout the United States, with 7.25% (95% CI, 6.43%-8.06%) of those visits (21 276 831) associated with either being currently prescribed an opioid or attaining one from the visit. Among pediatric patients, those aged 13 to 17 years were significantly more likely to have visits associated with opioid administration and/or prescription, with 16.20% (95% CI, 14.29%-18.12%) of individuals aged 13 to 17 years having an opioid prescribed compared with 6.59% (95% CI, 5.75%-7.43%) for those aged 3 to 12 years and 1.70% (95% CI, 1.42%-1.98%) for those aged 0 to 2 years (*P* < .001).

Significant differences in opioid prescription rates were observed across varying demographic factors. White participants reported an average opioid prescription rate of 8.11% (95% CI, 7.23%-8.99%) vs 5.31% (95% CI, 4.31%-6.32%) for nonwhite participants (*P* < .001). The likelihood of a visit being related to an opioid prescription was also statistically significant when comparing regions of the country, with visits occurring in the Northeast being associated with the lowest prescribing rate, having an average prescription rate of 4.69% (95% CI, 3.69%-5.70%) compared with 8.84% (95% CI, 6.82%-10.86%) for the West, the highest-prescribing region (*P* = .004). Patients using Medicaid for payment were less likely to receive an opioid than those using private insurance (5.47%; 95% CI, 4.79%-6.15% vs 9.73%; 95% CI, 8.56%-10.90%). The population density of the area where the individual received care did not appear to be associated with overall prescribing rates of opioids, with similar opioid prescribing rates between metropolitan and nonmetropolitan areas. Further details on cohort size and weighted and unweighted visit counts, as well as opioid prescription rates based on specific demographics throughout the cohorts, are available in Table 1.

**Table 1.** Emergency Department Visits for US Pediatric Patients, 2006-2015^a^

Variable	Total Visits		Visits With Opioid Prescription			*P* Value^b^
	Unweighted, No.	Weighted, No.	Unweighted, No.	Weighted, No.	Weighted, % (95% CI)	
All visits	69 152	293 528 632	4910	21 276 831	7.25 (6.43-8.06)	
Age, y						
0-2	22 515	95 677 472	403	1 626 298	1.70 (1.42-1.98)	[Reference]
3-12	30 213	129 048 017	1919	8 501 284	6.59 (5.75-7.43)	<.001
13-17	16 424	68 803 143	2588	11 149 249	16.20 (14.29-18.12)	<.001
Race						
Nonwhite	22 436	90 203 294	1138	4 793 140	5.31 (4.31-6.32)	<.001
White	46 716	203 325 338	3772	16 483 691	8.11 (7.23-8.99)	
Sex						
Female	32 727	13 943 0654	2320	10 091 754	7.24 (6.32-8.16)	.95
Male	36 425	154 097 978	2590	11 185 077	7.26 (6.42-8.10)	
Payment method						
Medicaid	35 602	149 785 565	1961	8 189 855	5.47 (4.79-6.15)	[Reference]
Private insurance	22 265	92 174 669	2104	8 972 087	9.73 (8.56-10.90)	<.001
Self-pay	4769	20 897 238	392	1 810 064	8.66 (7.03-10.29)	<.001
Other payment method	6516	30 671 160	453	2 304 825	7.51 (5.77-9.26)	<.001
Region						
Midwest	16 092	64 297 373	1104	4 411 876	6.86 (5.42-8.30)	[Reference]
Northeast	14 943	48 114 360	620	2 258 130	4.69 (3.69-5.70)	<.001
South	25 357	122 818 883	2078	9 453 757	7.70 (6.23-9.16)	.12
West	12 760	58 298 016	1108	5 153 068	8.84 (6.82-10.86)	.004
Metropolitan setting						
Metropolitan area	54 750	223 751 850	3888	16 127 742	7.21 (6.13-8.28)	.93
Nonmetropolitan area	8224	42 120 692	614	3 156 909	7.49 (4.22-10.77)	
Length of visit						
Not prolonged (≤240 min)	55 783	239 294 579	3728	18 814 630	6.79 (5.99-7.58)	[Reference]
Prolonged (>240 min)	10 229	38 880 272	1043	1 644 250	10.86 (9.48-12.23)	<.001
Unknown or missing data	3140	15 353 781	139	817 951	5.33 (3.07-7.58)	.01
Episode of care						
Follow-up visit to this emergency department	2540	9 869 291	211	807 085	8.18 (6.48-9.88)	[Reference]
Initial visit to this emergency department	52 155	233 274 583	3715	16 734 073	7.17 (6.36-7.99)	.17
Unknown or missing data	14 457	50 384 758	984	3 735 673	7.41 (6.04-8.79)	.37

^a^ All analyses account for the complex sampling design of the National Hospital Ambulatory Medical Care Survey.

^b^ *P* values calculated with Wald test.

The use of opioids in pediatric patients varies significantly based on the reported diagnosis related to the visit (Table 2). Visits associated with a bone fracture had the highest rate of opioid prescribing, with 41.30% (95% CI, 36.20%-46.40%) of all visits having an opioid prescribed. Pelvic pain, back pain, and other pain had the next-highest likelihood of having an opioid prescribed, with 21.21% (95% CI, 15.77%-26.65%), 20.99% (95% CI, 14.83%-27.15%), and 21.28% (95% CI, 16.11%-26.45%) of visits having an opioid prescribed, respectively. Injuries other than fractures were the least likely pain diagnosis to result in an opioid prescribed, with 9.75% (95% CI, 8.38%-11.13%) of visits having an opioid prescribed. Headaches, arthritis or joint pain, and abdominal pain had similar opioid prescribing rates per visit (16.57% [95% CI, 12.58%-20.57%], 15.27% [95% CI, 10.66%-19.88%], and 15.01% [95% CI, 12.29%-17.73%], respectively).

**Table 2.** Opioid Prescription Rates by Pain Diagnosis^a^

Pain Category	Total Visits		Visits With Opioid Prescription		
	Unweighted, No.	Weighted, No.	Unweighted, No.	Weighted, No.	Weighted, % (95% CI)
Not pain related	48 269	205 295 925	1718	7 425 946	3.62 (3.16-4.08)
Abdominal pain	2578	11 306 345	357	1 697 014	15.01 (12.29-17.73)
Arthritis or joint pain	635	2 632 174	97	401 903	15.27 (10.66-19.88)
Back pain	385	1 710 840	80	359 075	20.99 (14.83-27.15)
Fractures	2692	10 674 632	1043	4 408 498	41.30 (36.20-46.40)
Headache	919	4 186 025	158	693 811	16.57 (12.58-20.57)
Injury excluding fracture	12 305	51 975 509	1165	5 069 429	9.75 (8.38-11.13)
Other pain category	703	2 820 299	147	600 279	21.28 (16.11-26.45)
Pelvic pain	666	2 926 883	145	620 876	21.21 (15.77-26.65)

^a^ All analyses account for the complex sampling design of the National Hospital Ambulatory Medical Care Survey.

Logistic regression was used to analyze the association of demographic factors and pain diagnosis with the rate of visits during which an opioid was prescribed (Table 3). Age, race, sex, pain diagnosis, region of care, length of visit, and payment method were used as independent variables with opioid prescription as the dependent variable. Comparing the adjusted odds ratios (aORs) of the age groups showed that the aOR of a visit being associated with an opioid prescription more than doubled as age increased, using 13 to 17 years as the reference cohort. The age cohort of 0 to 2 years had an aOR of 0.14 (95% CI, 0.12-0.16) and the age group of 3 to 12 years had an aOR of 0.41 (95% CI, 0.38-0.45). All pediatric age groups had a lower rate of opioid prescribing than all adult age groups, with the 13 to 17 years group being roughly half as likely as the 18 to 24 years group to be prescribed an opioid; however, 16.20% (95% CI, 14.29%-18.12%) of those aged 13 to 17 years with an ED visit were still prescribed an opioid.

**Table 3.** Logistic Regression Analysis of Opioid Prescription Rates^a^^,^^b^

Characteristic	Adjusted Odds Ratio (95% CI)
Age, y	
0-2	0.14 (0.12-0.16)
3-12	0.41 (0.38-0.45)
13-17	1 [Reference]
Race	
Nonwhite	1 [Reference]
White	1.34 (1.19-1.50)
Sex	
Male	1 [Reference]
Female	1.03 (0.93-1.13)
Pain diagnosis	
Not pain related	1 [Reference]
Abdominal pain	2.69 (2.25-3.21)
Arthritis or joint pain	4.84 (3.61-6.50)
Back pain	4.27 (2.93-6.23)
Fractures	14.27 (12.60-16.16)
Headache	2.89 (2.21-3.77)
Injury excluding fracture	2.06 (1.83-2.32)
Other pain category	4.15 (3.12-5.52)
Pelvic pain	5.12 (3.80-6.92)
Payment method	
Private	1 [Reference]
Medicaid	0.74 (0.67-0.81)
Other or unknown	0.90 (0.77-1.07)
Self-pay	0.96 (0.82-1.13)
Region	
Midwest	1 [Reference]
Northeast	0.59 (0.50-0.69)
South	1.30 (1.13-1.50)
West	1.36 (1.14-1.62)
Prolonged emergency department visit	
Unknown or missing data	1 [Reference]
Not prolonged (≤240 min)	1.23 (0.92-1.65)
Prolonged (>240 min)	2.08 (1.54-2.82)

^a^ All analyses account for the complex sampling design of the National Hospital Ambulatory Medical Care Survey.

^b^ The model was simultaneously adjusted for the covariates listed.

Comparing opioid prescribing based on pain diagnosis revealed variable opioid prescribing rates dependent on the specific diagnosis. All non–pain diagnosis–related visits were used as the referent group, which had an overall opioid prescribing rate of 3.62% (95% CI, 3.16%-4.08%). Patients diagnosed with pain associated with a fracture had the greatest likelihood of being prescribed an opioid, with an aOR of 14.27 (95% CI, 12.60-16.16) when compared with the referent group. Pelvic pain and arthritis or joint–related pain were the next 2 pain diagnoses most likely to result in opioid prescribing, with aORs of 5.12 (95% CI, 3.80-6.92) and 4.84 (95% CI, 3.61-6.50), respectively. Visits associated with headaches and abdominal pain diagnoses had similar likelihood of opioid use, with aORs of 2.89 (95% CI, 2.21-3.77) and 2.69 (95% CI, 2.25-3.21), respectively.

Logistic regression analysis of payment method and opioid use demonstrated similar opioid prescribing rates among those who reported using private insurance, Medicare, and self-pay. Those using Medicaid as their primary payment method had a significantly lower likelihood of being prescribed an opioid, with an aOR of 0.74 (95% CI, 0.67-0.81) compared with those using private insurance. Comparing opioid prescribing in patients based on race revealed that white patients were more likely to be prescribed an opioid, with an aOR of 1.34 (95% CI, 1.19-1.50) compared with nonwhite patients. There was no difference in opioid prescribing between sexes, with female patients having an aOR of 1.03 (95% CI, 0.93-1.13) compared with male patients.

To analyze changes over time in opioid prescribing rates based on specific pain diagnoses, the original data set was separated into two 5-year cohorts, 2006 to 2010 and 2011 to 2015. Overall opioid prescription rates were significantly lower in the 2011 to 2015 cohort, decreasing from 8.23% (95% CI, 6.75%-9.70%) to 6.30% (95% CI, 5.44%-7.17%) (*P* < .001). Specifically, opioid prescribing rates for headaches, which had an overall prescribing rate of 16.6%, decreased from 21.93% (95% CI, 14.96%-28.90%) to 12.51% (95% CI, 7.54%-17.47%) (*P* = .008). The opioid prescribing rates for non–fracture-related injuries, abdominal pain, and non–pain-related visits also decreased over this time (Table 4). Additionally, opioid use in non–pain-related visits decreased from occurring in 4.07% of visits to 3.18% of visits. Other diagnoses, including pelvic pain, fractures, back pain, and arthritis or joint pain, did not have a statistically significant reduction in opioid prescribing over the same period. Further details are available in Table 4.

**Table 4.** Pain-Related Opioid Prescription Trends Over Time^a^

Pain Category	2006-2010					2011-2015					*P* Value^b^
	Total Visits		Visits With Opioid Prescription			Total Visits		Visits With Opioid Prescribed			
	Unweighted, No.	Weighted, No.	Unweighted, No.	Weighted, No.	Weighted, % (95% CI)	Unweighted, No.	Weighted, No.	Unweighted, No.	Weighted, No.	Weighted, % (95% CI)	
All opioid prescriptions	40 698	144 083 899	3094	11 855 012	8.23 (6.75-9.70)	28 454	149 444 733	1816	9 421 819	6.30 (5.44-7.17)	.005
Not pain related	28 436	100 369 050	1077	4 086 315	4.07 (3.25-4.89)	19 833	104 926 875	641	3 339 631	3.18 (2.63-3.74)	<.001
Abdominal pain	1392	5 060 459	217	894 048	17.67 (12.97-22.36)	1186	6 245 886	140	802 966	12.86 (9.48-16.24)	.01
Arthritis or joint pain	392	1 435 996	63	257 757	17.95 (10.27-25.62)	243	1 196 178	34	144 146	12.05 (7.01-17.09)	.17
Back pain	206	750 832	43	173 636	23.13 (13.10-33.15)	179	960 008	37	185 439	19.32 (11.08-27.55)	.52
Fractures	1709	5 904 487	667	2 494 409	42.25 (34.72-49.77)	983	4 770 145	376	1 914 089	40.13 (33.16-47.10)	.41
Headache	486	1 806 646	96	396 193	21.93 (14.96-28.90)	433	2 379 379	62	2 97 618	12.51 (7.54-17.47)	.008
Injury excluding fracture	7284	25 950 359	750	2 922 544	11.26 (9.03-13.49)	5021	26 025 150	415	2 146 885	8.25 (6.75-9.75)	<.001
Other pain category	403	1 353 625	88	301 074	22.24 (14.13-30.35)	300	1 466 674	59	299 205	20.40 (13.56-27.24)	.66
Pelvic pain	390	1 452 445	93	329 036	22.65 (14.52-30.78)	276	1 474 438	52	291 840	19.79 (12.30-27.29)	.54

^a^ All analyses account for the complex sampling design of the National Hospital Ambulatory Medical Care Survey.

^b^ *P* values calculated with Wald test.

## Discussion

The potentially addictive nature of opioids, coupled with their widespread use, has led to major discussions throughout the US health care system regarding the safety and effectiveness of using opioids overall. Opioid use in pediatrics has been met with additional clinical and ethical questions, with the latest CDC recommendation for opioid use in chronic pain suggesting that opioid use in pediatrics, particularly adolescents, be avoided whenever possible.^15^ Although much of the focus of better management of opioid use has focused on the adult population, several researchers have expressed concerns regarding the overprescription of opioids and excessive prescriptions for opioids in pediatric patients. The prescribing of opioids to pediatric patients is of particular concern because of this group’s increased risk of addiction and the potential long-term effects related to opioid abuse in pediatric patients.^8,16^

The use of opioids in pediatric patients in certain clinical circumstances is appropriate and necessary. The intent of this review is to evaluate the trends of use in a number of clinical diagnoses and evaluate patterns of use and inconsistencies, if any, in the use of opioids. For instance, the use of opioids to treat pain associated with an injury resulting in a bone fracture is likely appropriate, but an evaluation of patient-specific factors associated with prescribing trends in patients with fractures helps to assess consistency in use.

However, despite the importance of understanding the prescribing trends related to pediatric opioid use, very little research has been done in this area. The analysis presented here reveals that a large portion of the pediatric population continues to be prescribed opioids when being treated in EDs, with significant variations related to race, age, diagnosis, payment method, and region of the country where the patient visit occurred.

As anticipated, age had a significant association with the use of opioids, both in pediatric patients and adults. Those aged 13 to 17 years were significantly more likely to have visits associated with opioid use (16.20% of visits) compared with those aged 3 to 12 years (6.59% of visits) and 0 to 2 years (1.70% of visits). Although the overall rate of opioid prescribing for pediatric patients was lower than for adults, the fact that more than 16% of all visits for those aged 13 to 17 years have an opioid associated with them suggests that overall opioid use is also relatively high in pediatric patients. The rate of opioid prescribing related to headaches in this study was 16.6%, which was significantly lower than the previously reported rate of 46%.^17^ This difference in reporting is likely due to the nature of the data provided, with the data restricted to a single visit, vs previous reports reviewing all prescription drug use over a full year. Additionally, previous research focused on opioid use in the treatment of headaches, specifically in patients aged 13 to 17 years, and this study includes analysis of use across all patients aged 17 years and younger.

Time-lapse analysis does show a reduction in overall opioid use comparing 2006 to 2010 with 2011 to 2015. This reduction suggests that the scrutiny of the safety of opioid use overall and particularly in pediatric patients has resulted in prescribers reevaluating their use of opioids overall. This reduction in use is primarily driven by reductions in opioid use in specific pain diagnoses, including headache, abdominal pain, and non–fracture-related injuries. The overall reductions were also partially the result of reduced opioid use in non–pain-related visits, which decreased from occurring in 4.07% of visits to 3.18%. The lack of change over time in the use of opioids for other pain diagnoses, particularly back and pelvic pain, suggests that alternative treatment options need to be further evaluated or encouraged among health care professionals. The use of opioids in back pain is of significant concern because growing research suggests that chronic use of opioids to treat back pain is not effective for both pain and function and is associated with significant safety concerns.

Our findings indicate that patient visits in the western region of the country (Alaska, Arizona, California, Colorado, Hawaii, Idaho, Montana, Nevada, New Mexico, Oregon, Utah, Washington, and Wyoming) are significantly more likely to be prescribed an opioid, while patients visits in the Northeast (Connecticut, Maine, Massachusetts, New Hampshire, New Jersey, New York, Pennsylvania, Rhode Island, and Vermont) are associated with the lowest likelihood of an opioid being prescribed despite a lack of significant difference in pain-related diagnosis in these region. Factors affecting the regional variability in opioid prescribing in pediatric patients need to be further evaluated to better understand what may be driving the overall prescribing patterns in each region, particularly those with higher opioid prescribing rates.

Race additionally had a significant association with overall prescription trends, with white patients having a significantly increased likelihood of being prescribed an opioid compared with nonwhite patients. These findings are similar to previous research that has indicated significant racial differences in prescription patterns of opioids in adult patients.^18,19,20^ When holding constant for pain diagnosis, white patients were significantly more likely to receive an opioid. For instance, white patients reporting abdominal pain were almost twice as likely to receive an opioid than their nonwhite counterparts. While there are numerous potential explanations for this difference, further subset analysis is needed to better evaluate the use of opioids based on race to determine the association of pain scale reports, age at time of visit, other patient-specific factors, and prescriber-specific factors with opioid prescribing across racial and ethnic groups.

Similar to previous research, individuals whose primary payment method reported was state Medicaid had a lower likelihood of being prescribed an opioid.^21^ This raises questions regarding the biases that may exist in prescribers when treating patients covered by state Medicaid programs, which appears to be true in pediatric patients as well as adults. This study’s analysis found that, unlike previous studies in adults, prescribing rates were similar between sexes.

The relationship between opioid use rates in pediatric patients and pain diagnoses suggests that different pain-related diagnoses are viewed by prescribers as more appropriate than others for opioid use. As expected, pain related to fracture injuries had the highest rate of opioid prescribing, which remained consistent when comparing 2006 to 2010 with 2011 to 2015. The relatively high use of opioids in pelvic pain and back pain, which also lack a statistically significant reduction over time, suggests additional evaluation is necessary regarding prescribers’ attitudes toward treatment options in these specific pain-related diagnoses. Although the percentage of visits for headache, abdominal pain, non–fracture-related injuries, and non–pain-related diagnoses associated with opioid use has decreased, the continued use of opioids suggests additional opportunity for further evaluation and prescriber outreach to ensure appropriate and consistent prescribing of opioids. Each of these pain diagnoses have limited need for the use of opioids, and the reduction of use over time is a positive trend; however, more significant reductions of opioid use in these clinical circumstances would be appropriate.

### Limitations

Limitations of this study include its retrospective nature and the general limitations of using a cross-sectional data set. Owing to this limitation, we can only report the findings of the analysis and are not able to draw conclusions about the causative nature of factors associated with opioid prescribing patterns. Additionally, this study is not able to determine which factors may have been associated with a reduction in the use of opioids for specific pain diagnoses. Many factors may need to be further explored to understand their role in opioid prescribing patterns. In addition, a number of comorbidities and preexisting diseases may affect the choice of drug for pain management. For example, opioid therapy is generally the first-line approach for moderate to severe pain control, alone or in combination with other medications, in patients with active cancer, particularly when a patient is neutropenic. The presented study is not able to evaluate patients’ comorbidities or determine their potential effects on opioid prescribing.

## Conclusions

The findings of this study suggest the overall use of opioids in pediatric patients in EDs has declined since 2010; however, further research is warranted given the remarkable variability in opioid prescribing rates based on patient-specific demographic characteristics and pain-related diagnoses. Although the use of opioids is indicated for specific pain-related visits, it is important to keep prescribing consistent across all health care professionals regardless of patient demographic characteristics. The lack of consistency in prescribing opioids observed in this study also indicates the need to develop a more standardized opioid use guideline in pediatrics, which can help guide prescribers to make informed decisions when considering opioids for the treatment of patients younger than 18 years.
